# A multivariate cell-based liquid biopsy for lung nodule risk stratification: Analytical validation and early clinical evaluation

**DOI:** 10.1016/j.jlb.2025.100313

**Published:** 2025-07-26

**Authors:** Jason D. Berndt, Fergal J. Duffy, Mark D. D'Ascenzo, Leslie R. Miller, Yijun Qi, G. Adam Whitney, Samuel A. Danziger, Anil Vachani, Pierre P. Massion, Stephen A. Deppen, Robert J. Lipshutz, John D. Aitchison, Jennifer J. Smith

**Affiliations:** a PreCyte, Inc., Seattle, WA, USA; b Seattle Children's Research Institute, Seattle, WA, USA; c Institute for Systems Biology, Seattle, WA, USA; d Pulmonary, Allergy, and Critical Care Division, University of Pennsylvania, Philadelphia, PA, USA; e Thoracic Program, Vanderbilt-Ingram Cancer Center, Vanderbilt University, Nashville, TN, USA; f Department of Surgery, Tennessee Valley Healthcare System, Veterans Affairs, Nashville, TN, USA; g Department of Thoracic Surgery, Vanderbilt University Medical Center, Nashville, TN, USA; h Department of Global Health, University of Washington, Seattle, WA, USA

**Keywords:** Liquid biopsy, Biosensor, Multivariate classifier, Lung cancer screening, Indicator cell assay platform (iCAP), Hypoxia, Precision oncology

## Abstract

**Background:**

The Indicator Cell Assay Platform (iCAP) is a novel tool for blood-based diagnostics that uses living cells as biosensors to integrate and amplify weak, multivalent disease signals present in patient serum. In the platform, standardized cells are exposed to small volumes of patient serum, and the resulting transcriptomic response is analyzed using machine learning tools to develop disease classifiers.

**Methods:**

We developed a lung cancer-specific iCAP (LC-iCAP) as a rule-out test for the management of indeterminate pulmonary nodules detected by low-dose CT screening. This included assay parameterization, analytical reproducibility testing, and selection of a fixed 85-gene feature set for future clinical validation and regulatory development. Clinical performance was estimated using a prospective-specimen-collection, retrospective-blinded-evaluation (PRoBE) study design comprising 176 samples. Classifier variants were trained by nested cross validation using subsets of the 85 genes, and selected variants were evaluated by temporal blind validation using 39 control and 40 case samples (72 % Stage I, 22 % Stage II cancer).

**Results:**

The assay showed excellent reproducibility across various conditions and cell lineages, and case versus control transcriptomic signals were enriched for hypoxia-responsive genes, consistent with known lung cancer biology. Two models demonstrated discriminative ability in blind validation, one with AUC = 0.64 (95 % CI: 0.51–0.76). Post hoc integration with CT imaging features yielded a combined model with 90 % sensitivity, 64 % specificity, and 95 % negative predictive value at 25 % prevalence, suggesting clinical utility and surpassing performance of existing rule-out tests.

**Conclusion:**

This study establishes the analytical reproducibility and biological relevance of the LC-iCAP. While clinical validation is preliminary, the results support the assay's potential utility in lung nodule management. The study introduces a new paradigm of using scalable and cost-effective cell-based biosensor assays for liquid biopsies. With a multivariate readout, the platform is amenable to precision medicine applications such as multi-cancer early detection.

## Highlights

• Introduces a novel liquid biopsy approach that uses standardized cells as biosensors.
• Yields multivariate classifiers useful for precision oncology.
• Has high analytical reproducibility.
• Has promising performance in reducing false positives in CT-based lung cancer screening.
• Hypoxia-related readout suggest broader utility in cancer detection.

## Teaser

The LC-iCAP uses cells as biosensors to turn complex blood signals into gene expression profiles. Machine learning then identifies patients at higher risk for lung cancer.

## 1 Introduction

**Fig. 1:**
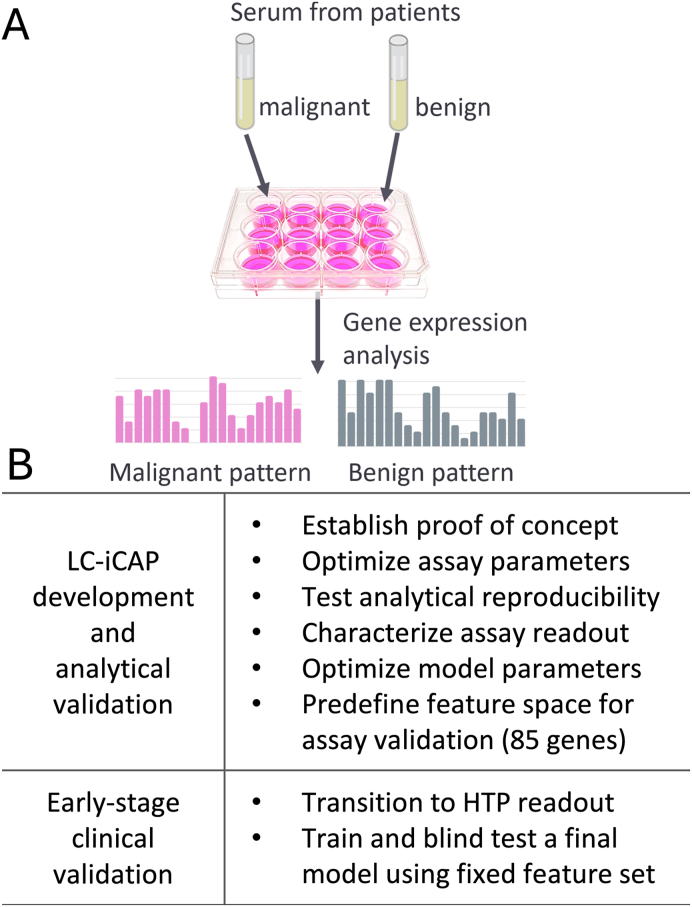
*A*, The iCAP for blood-based diagnostics. Standardized cells are exposed to serum from patients. Gene expression readout of the cells is used to develop machine learning models to predict disease. *B,* Two stages of LC-iCAP development. *HTP*, high throughput.

The indicator cell assay platform (iCAP) [2] is a novel approach that aims to overcome the low signal-to-noise ratio associated with direct measurement of blood biomarkers by using cultured cells as biosensors. Developing an iCAP involves exposing standardized, cultured cells to small volumes of serum from case and control participants, measuring a multivariate gene expression response of the cells, and using subset of the features for machine learning-based disease classification (Fig. 1A). This strategy takes advantage of the natural signal amplification and integrative sensing capacity of living cells, broadening the detectable analyte space while delivering a standardized gene expression readout. For deployment, the cell-based assay can be implemented in scalable workflow using targeted high throughput platforms such as NanoString nCounter Dx or Ion AmpliSeq.

Pulmonary nodules identified by low-dose computed tomography (CT) screening pose a major clinical challenge. While patients with malignancy risk below 5 % or above 65 % have relatively clear management pathways, the majority fall into an intermediate-risk zone (5–65 %) where decisions are uncertain. Many of these patients undergo invasive and costly diagnostic procedures despite having benign disease [3,4]. Non-invasive rule-out tests with a negative predictive value (NPV) of ≥95 % are needed to reclassify risk to <5 %, reduce unnecessary interventions, and improve patient outcomes [[5], [6], [7]]. While two commercial blood-based rule-out tests exist [8,9], their standalone performance is weak and their integration with imaging yields low specificity. A third test offers better performance, but requires invasive bronchoscopy, limiting its utiltiy [10].

Here, we describe the development and validation of LC-iCAP, a lung cancer-specific iCAP assay designed to address this gap. LC-iCAP was developed for the early detection of non-small cell lung cancer (NSCLC) in patients with indeterminate pulmonary nodules (IPNs). Assay development included protocol optimization, analytical reproducibility testing, and selection of a fixed 85-gene panel to support future clinical validation studies and standardized clinical deployment, consistent with approaches established in the development of multigene assays for breast cancer [11,12]. Clinical performance was evaluated in a prospective-specimen-collection, retrospective-blinded-evaluation (PRoBE) study of 176 serum samples. An LC-iCAP classifier was trained using the fixed gene feature set on samples from current former smokers with IPNs and validated using a retrospective-blind-temporal design. In validation, the assay achieved an AUC of 0.64 (95 % CI, 0.51–0.76). When integrated post hoc with CT data, the assay yielded 90 % sensitivity, 64 % specificity, and an NPV of 95 %, assuming 25 % disease prevalence [13,14]. These results compare favorably to existing blood-based rule-out tests and suggest that LC-iCAP may deliver actionable results to a broader population.

Beyond lung nodules, the iCAP is a flexible, multivalent biosensing system that supports high-throughput implementation and is orthogonal to existing diagnostic approaches. Its architecture and multivariate readout are well suited for future applications in multi-cancer early detection (MCED), minimal residual disease monitoring, combinatorial diagnostics, and disease stratification for precision oncology.

## 2 Methods

**Participants and specimen characteristics.** The study used archived serum samples collected prospectively from adult patients from non-vulnerable populations performed with institutional review board (IRB) approval (WCG IRB study 1283522). Banked specimens and clinical data used in this study were from subjects enrolled in the following previously IRB-approved studies: “Molecular Predictors of Lung Cancer behavior,” (NCT00898313, Vanderbilt University), “Gene-Environment Interactions in Lung Cancer” (IRB 806390, University of Pennsylvania), and “A Case Control Study of Smokers and Non-Smokers” (IRB 800924, University of Pennsylvania). The study consent forms had provisions allowing use of their samples for future research purposes. Patient identifiable information was not provided to the research team and was not used in this study. All identification numbers used in the manuscript are not known to anyone outside the research group.

Patient attributes and serum characteristics are shown in Table I and Data Files 1 and 4. Serum samples were collected at the time of CT scan from patients who were representative of the target population with the intention of using the serum for developing liquid biopsies in the future. All samples were collected within 3 months of the CT scan (and diagnostic biopsy, if performed) and prior to any invasive procedure, including surgical lung biopsy. All malignant nodules were diagnosed by follow-up pathological diagnosis; all were non-small cell lung cancer (NSCLC) except for 3 small cell lung cancers. Benign nodules were diagnosed by either biopsy with a definitive benign histological diagnosis or by 2 or more years of follow-up with serial imaging. All patients had no known other cancers at time of screening and no previous cancer in the five years preceding the blood draw excluding previous skin cancers treated with surgery only (no radiation or chemotherapy). Serum samples were collected using the protocol recommended by the early detection research network (EDRN) [15], stored at −80 °C or below after collection, and unless otherwise stated, thawed once prior to LC-iCAP analysis. Case and control subjects were selected randomly from the biobank and their serum was used for developing the LC-iCAP.

**Table 1:** Samples used for LC-iCAP optimization and validation. Patients had IPNs identified by CT scan that were classed as malignant or benign by either diagnostic biopsy or resection or by 2 or more years of serial imaging. Patents had no other known cancer at the time of CT scan. Cohorts 1–4 were from Vanderbilt University. A 5th cohort from University of Pennsylvania was used for sample quality analysis only (see Methods). LC-iCAP optimization used cohorts 1 and 2 and a subset of cohort 3. LC-iCAP validation used cohorts 1–3 for model training and cohort 4 for blind validation. Cohort 4 was temporally independent as it was collected 9 years later and assayed 1 year later than the other samples. *IPN*, indeterminate pulmonary nodule; *CT*, low dose computed tomography.

Class	Description	cohort 1	cohort 2	cohort 3	cohort 4 BLIND
Serum from patients with non-calcified IPNs identified by CT scan between 5 and 30 mm in diameter (95 % > 7 mm)	Case: patients with malignant nodules	6	53	50	40
	Control: patients with benign nodules	6	50	50	40
subtotal		12	103	100	80
Total sample count		295			

**Study design.** LC-iCAP development was done in two stages (Fig. 1B). The first stage, development and analytical validation, included optimization of assay parameters, testing of analytical reproducibility, characterization of the assay readout, optimization of model parameters, and fixing a feature set of 85 transcripts for the next stage. The second stage, early-stage clinical validation, included transition to a high-throughput readout, nested cross validation, and testing fully specified models using a blind test set. This validation study had a prospective-specimen-collection, retrospective-blinded-evaluation (PRoBE) design and reporting in the methods and results sections follows recommendations of TRIPOD (Transparent Reporting of a multivariable prediction model for Individual Prognosis Or Diagnosis) [16]. Throughout both stages, standard controls were used to identify technical failures and low-quality samples, which were excluded.

**Sample processing.** Sample processing batch structure is described in Data files 1 and 4. Each cohort was shipped separately and assayed by LC-iCAP in batches of 20–50 samples. Each experimental batch had ∼1:1 ratio of case and control samples, which were roughly balanced for patient gender, age and smoking history. A pair of standard controls were included on each 12-well plate for quality control (QC) consisting of either technical replicates of a reference serum sample from an unaffected male patient (cohorts 1–2), a pair of case and control pooled serum controls (cohort 3), or a pair of DMOG and PBS chemical controls (cohort 4 and University of Pennsylvania samples). LC-iCAP gene expression was measured by either RNA-seq or NanoString Plexset.

**Quantification of hemolysis of serum samples.** Prior to LC-iCAP analysis, thawed patient serum was evaluated for the breakdown of red blood cells (hemolysis), which has known interference with clinical biochemical tests [17]. Blind samples were visually compared to a reference card [18] by a first scientist and given a score on a gradient of increasing hemolysis from 1 to 7. In addition, samples were photographed with an iPhone on a white background and later evaluated by a second scientist using the same approach. Sample ratings were averaged and round to the nearest integer. For all modeling conducted after the pilot study, all samples with average scores greater than 3 (>50 mg/dL of hemoglobin) were omitted from the study. Hemolysis scores are shown in Data files 1 and 4.

**Analytical parameters of LC-iCAP assay.** Unless otherwise indicated, the following standard protocol was used: 2 x 10^6^ lung epithelial cell indicator cells (16HBE14o- (16HBE)) were thawed and plated in a T75 flask in RPMI with 10 % FBS (complete medium). After 2 d cells were dissociated with 0.25 % trypsin-EDTA for 10 min at 37 °C and plated at 30,000 cells/cm^2^ in 12-well Eppendorf moat plates in complete medium. After 24 h, cells were rinsed once with RPMI and incubated for 24 h with in 1 mL RPMI with 5 % patient serum. Media was removed, lysis buffer was added, and cells were stored at −80 °C for up to two weeks before RNA isolation. For cohorts 1 and 2 used in assay optimization, total RNA was isolated manually using RNeasy Mini Kit (Qiagen), for cohorts 3–4 and University of Pennsylvania samples, RNA isolation was automated using a MagMax mirVana kit (Invitrogen A27828) on either a KingFisher Flex or a Kingfisher Duo Prime as per the manufacturer's recommendation. RNA was eluted in 100 μL of elution buffer, quantified using a Qubit (Qubit RNA BR Assay Kit), and stored at −80 °C. Transcript abundance levels were quantified using either RNA-Seq or NanoString as described below. For cohort 3, cells were passaged an additional time before initiating the experiment.

**LC-iCAP proof-of-concept study.** To establish assay feasibility, LC-iCAP RNA-seq data for cohort 1 was used to train a model for lung cancer detection and tested on cohort 2. To identify model features, raw counts from cohort 1 data were used for differential expression analysis and identified 239 differentially expressed genes (DEGs) (Benjamini-Hochberg false discovery rate (FDR) < 0.05). Next, to develop the LC-iCAP classifier, all RNA-seq count data from cohorts 1 and 2 were normalized using the DESeq2 rlog transformation and a series of random forest classifiers (R: randomForest package) were parameterized on cohort 1 using increasing numbers of DEGs with the lowest FDR scores as features (including 5, 10, 20, 25, 50, 75, and 100 genes). The 103 samples of cohort 2 were used to test the model performance.

For hierarchical clustering of LC-iCAP data from cohorts 1 and 2, clustering was performed based on the expression of the top 20 differentially expressed in LC-iCAP RNA-seq data from cohort 1. The analysis utilized DEseq2 rlog-transformed counts from the LC-iCAP RNA-seq data, normalized to the mean expression of the benign samples of the same iCAP experimental batch and gene rank was based on median absolute deviation. Three outlier samples were identified and removed (all from the benign class).

**RNA-Seq analysis of LC-iCAP RNA.** 100–650 ng of total RNA per sample was used for automated library preparation and RNA sequencing (RNA-seq) performed by either Covance (cohorts 1–2) or Azenta Life Sciences (formerly Genewiz) (cohort 3). Strand-specific library prep was performed with PolyA selection using TruSeq RNA library Prep Kit (Illumina) with unique dual indices (IDT) and resulting DNA was sequenced on a HiSeq 4000 (Illumina) with paired end 150 bp reads. RNA-seq data were processed using a custom workflow including adapter read trimming using *trimmomatic* [19], genome reference alignment to HG37 (pilot study) or HG38 using *STAR* [20]; and gene-level transcript quantification using R-featureCounts [21]. ERCC spike-ins and genes with mean absolute counts <10 were removed. Quality was assessed using MultiQC, dupRadar and GATK estimate of library complexity. Where indicated, counts were adjusted to correct GC bias using the FQN [22] or CQN [23] determined by the R-EdgeR package [24]. Differential expression analysis was done using R-DESeq2 [25]. GSEA analysis was done using the R-fgsea package in combination with the 50 Hallmark pathway modules from MsigDB [26].

For proof of concept analyses, read duplicates were removed by using –ignoreDups setting in R:featureCounts. For all other assay optimization analyses, the data were normalized for heteroskedasticity by variance stabilizing transformation (VST) using the R-DESeq2 package and for inter-iCAP batch variation using removeBatchEffect from the R-limma package [27]. Outlier samples were identified using robust principal components analysis (ROBPCA) implemented in the R-rrcov package [28].

**Fig. 2:**
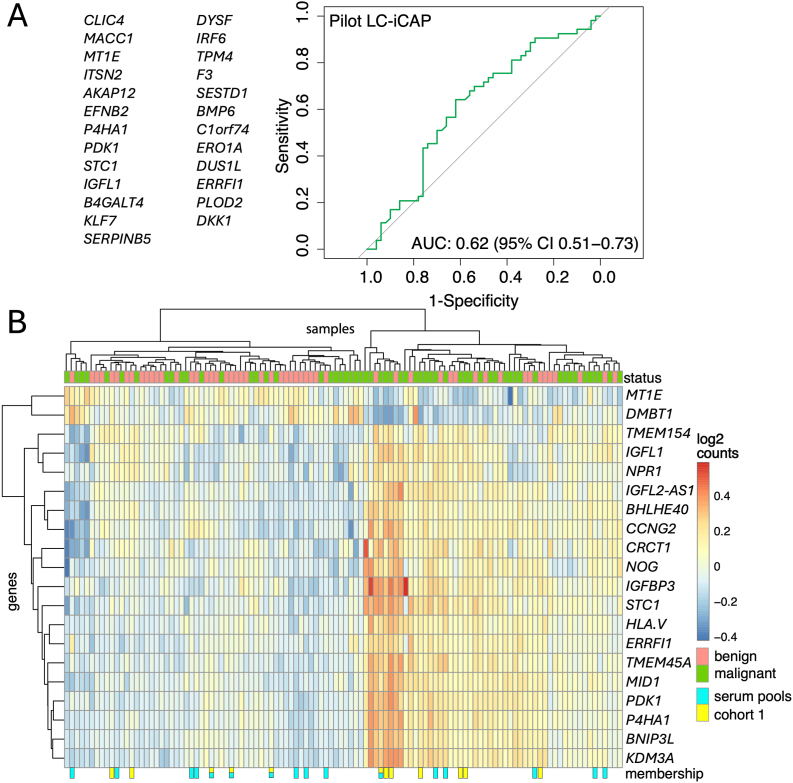
Proof of concept of LC-iCAP. A. ROC curve showing performance of a pilot LC-iCAP model trained on cohort 1 (n = 12) and tested on held-out cohort 2 (n = 103) using 25 gene expression features (left). B. Hierarchical clustering of cohorts 1 and 2 based on LC-iCAP gene expression shows grouping of samples by class. Clustering was based on the top 20 differentially expressed genes in cohort 1 based on median absolute deviation. Dendograms show two distinct sample clusters broadly separating case and control samples (top). 3 outlier samples were omitted. Samples used to make the biological standards (serum pools) and the 12 samples of cohort 1 are marked in cyan and yellow, respectively (bottom).

**Development of LC-iCAP standard controls.** Serum pools were prepared for each case and control class and used as a pair of biological standards to monitor assay performance. Each pool consisted of a mix of serum from 8 different subjects selected from cohorts 1 and 2 based on availability and class separation in hierarchical clustering analysis of LC-iCAP-RNA-seq data (Fig. 2B). Each serum pool was made by thawing aliquots of serum from each of 8 subjects, pooling, mixing, aliquoting the serum, and then flash freezing in liquid nitrogen before storing at −80 °C.

Dimethyloxalylglycine (DMOG) and PBS were used as a pair of chemical standards to monitor assay performance. DMOG was selected because it known to transcriptionally activate hypoxia-responsive genes, and thus mimics plasma from patients with lung cancer in the LC-iCAP. To develop the control, we first characterized the response of indicator cells to 6 concentrations of DMOG (0, 0.025, 0.05, 0.1, 0.25, 0.5 mM; Cayman Chemical) by measuring the gene expression readout of the 74-gene development gene set (Data file 2A) using NanoString. Responsive genes were identified as those whose expression fit a linear model as a function of DMOG concentration (p-value <0.05) (data not shown). From these data, we selected 0.25 mM as the standard DMOG concentration for monitoring assay performance; for most responsive genes, this condition was in the linear range of the model and had a similar magnitude of responsiveness compared with patient serum. DMOG was resuspended at 50 mM in PBS, aliquoted and stored at −20 °C and thawed on ice before use.

**Optimization of LC-iCAP experimental parameters and LC-iCAP reproducibility testing.** The pooled serum standards described above were used for QC and for reproducibility and optimization studies. For each experiment, 4 technical replicates of each case and control serum pool were analyzed for each LC-iCAP parameter and the number of significantly DEGs (FDR <0.1) for each configuration were compared. Differential expression was measured by analysis of the LC-iCAP development gene panel using NanoString nCounter® technology and/or by RNA-seq using HiSeq4000™ (Illumina). Configurations tested were various serum concentrations (1 %, 5 %, 10 %, and 20 %), serum incubation times (6 h, 24 h), and 4 cell types (16HBE, A549, MRC5, and Nuli-1). Reproducibility testing included comparison across 3 different 16HBE expansion batches and across different days, and different detection platforms.

**Western blot analysis.** Samples were processed in the LC-iCAP using standard parameters and protein was isolated and quantified using a BCA kit (Pierce). Equal protein for each sample were loaded on 12- or 15-well NuPage 4–12 % Bis-Tris gels with Thermo PageRuler Plus prestained protein ladder and analyzed by western blotting according to LICOR Odyssey recommendations using PVDF membrane (Immobilon-FL) and probing with rabbit anti-HIF1a (D157W) XP® (or rabbit mAB HIF-2a (D9E3)) and mouse anti-beta-actin primary antibodies followed by LICOR NIR secondary antibodies. All primary antibodies were from Cell Signaling Technologies. Membranes were scanned on a LICOR Clx Odyssey imaging system, proteins were quantified using LiCOR Image Studio Lite and data analysis was done in Excel. Western blot images are shown in Fig. S5.

**Sample and Gene Filters for Comprehensive Modeling Study in**Fig. 5**.** Data from 165 samples across cohorts 1–3 were partitioned into training and validation sets, and quality filters were applied, resulting in 137 samples for modeling (Table 1, Data file 1). These data were used for a comprehensive modeling study including 13 different feature selection methods x 8 combinations of 3 optional filters. The three sample filters were: 1) samples with predicted forced expiry volume (FEV) < 50 % (based on data showing that low lung function affects LC-iCAP readout (Fig. S6)), 2) samples from never-smokers, and 3) samples from a low-quality RNA-seq batch (identified by QC analysis with assay standards; Fig. S3). The 13 feature selection methods are described in Data file 3 and are based on 3 approaches to identify case versus control differential expression in the training set: 1) Analysis of DEGs across samples from all batches, 2) analysis of DEGs within each individual experimental batch, and 3) GSEA of individual experimental batches.

**Gene sets for targeted analysis by NanoString.** Gene-specific capture probes for NanoString nCounter analysis were synthesized by Integrated DNA Technologies (IDT). The NanoString development gene set was used for LC-iCAP parameter optimization and reproducibility studies. The set consisted of 96 genes including 74 features with case versus control differential expression in cohort 1 selected for detecting the LC-iCAP readout and 7 housekeeping genes for normalization (Data file 2A). The NanoString deployment gene set was used for training and testing the final LC-iCAP models in the assay validation study using a NanoString Plexset readout. The gene set consisted of 95 genes including 85 candidate features for modeling (66 selected in the model optimization (Figs. 5) and 19 of the 25 genes from the initial proof of concept model not already in the list), 1 control gene responsive to DMOG but not case serum and 9 housekeeping genes for normalization (Data file 3D).

**NanoString Plexset™ analysis of LC-iCAP RNA.** Gene expression analysis of total RNA samples from the LC-iCAP was performed using NanoString Plexet™ technology, a direct detection approach for multiplexed gene expression analysis of up to 96 samples per run with no PCR amplification. For this analysis, the ‘deployment gene set’ was used consisting of 85 target genes and 8 candidate housekeeping genes (Data file 3). Data were analyzed using an nCounter® Analysis System by the Genomics Resources Center at Fred Hutchinson Cancer Research Center following manufacturers recommendations. First, probe hybridization was performed in solution where gene-specific capture probes and reporter probes attached to fluorescent barcodes (code sets) were used for detection of each of 96 transcripts in each sample (see Data file 3). Each reaction included LC-iCAP RNA from patient samples (140 ng) or from DMOG or PBS controls (100 ng), amounts that were pre-optimized in a calibration experiment to avoid saturation. Next, samples were pooled in groups of 8 and loaded onto an nCounter Prep Station for automated excess probe removal and binding of the probe-target complexes on the surface of the cartridge by streptavidin-biotin linkage to the capture probes. Cartridges were placed in the nCounter Digital Analyzer for data collection, where molecules of RNA were counted by using the target specific “color codes” generated by a string of six fluorescent spots on each reporter probes.

LC-iCAP analysis was done using our standard approach except serum samples for cohorts 1–2 were thawed twice before analysis. LC-iCAP RNA samples were processed on 7 plates with up to 96 samples per plate (Data file 4). Each plate included 8 positive controls composed of *in vitro* transcribed RNA transcripts and corresponding probes, and eight negative controls consisting of probes with no sequence homology to human RNA for lower limit of detection analysis. One positive and one negative control were used for each of the 8 multiplexed samples in each lane. Analysis of training set samples (plates 1–6) and blind test set samples (7th plate) were separated by one year using two different code set manufacturing lots. To facilitate code set lot normalization in downstream processing, technical iCAP replicates of a calibration sample were run with code set lot 1 (training samples) and code set lot 2 (blind test samples).

Processing of raw Plexset data was performed using nSolver software following manufacturer's recommendations (MAN-C0019-08) as specified below. Data points below the lower limit of detection were floored to a threshold of 20 counts. Next correction for technical variation was done by housekeeping (HK) gene normalization, for which the expression value of each gene was divided by the geometric mean of 5 stably expressed housekeeping genes (*ABCF1*, *FCF1*, *GUSB*, *POLR2A*, and *SDHA*) in the same sample. Next, within-plate code set calibration was performed to normalize the 8 different sets of barcodes used for each of the 8 rows. Finally, code set lot calibration was done to normalize lot 1 (training samples in plates 1–6) and lot 2 (blind test samples in plate 7) using the “batch normalization” tool of nSolver. Prior to using Nanostring data for modeling, raw counts were log_2_ transformed, and outlier removal was performed using ROBPCA [29] analysis of housekeeping genes only.

**NanoString Plexset reproducibility.** To investigate the effectiveness of the plate normalization (within training samples (plates 1–6)) and code set lot calibration (between training and blind test samples), hierarchical clustering was performed on the chemical and biological controls in the LC-iCAP Plexset data. While good plate to plate reproducibility was observed, code set lot mis-calibration was identified which resulted in differential expression of in the LC-iCAP between control samples of the training and test sets (p-value <0.005) (compare lot 1 versus lot 2 in Fig. S9, Data file 4).

**Modeling.** Generalized linear models (GLMs) were implemented in the R-glmnet package [30]. RF models were implemented in the R-caret package [31] using mtry values that were automatically selected for each seed using the default settings. For the proof-of-concept model, RF models were trained by leave-one-out cross validation with 10 random seeds and tested on a held-out independent validation set. For the large-scale modeling study in Fig. 5, RF models were training set by leave-one-out cross validation with 20 random seeds and 50 resampling iterations and tested on a held-out independent validation set. Each model had a maximum 20 gene features selected based on variable importance score and all models were repeated with downsampling of the training set to balance the number case and control samples within each experimental batch to assess model robustness. Top models were selected based on performance on the validation set (using either AUC or specificity at a fixed sensitivity of ≥95 %).

For modeling in the final assay validation study with LC-iCAP NanoString Plexset data, RF or GLM models were trained and validated using 5-fold nested cross-validation with 50 subsamples per seed, implemented using R-nestedcv framework [32]. Cross-validation was repeated 10 times with different random seeds, and RF models used 500 trees. Features were log_2_ normalized iCAP gene expression values, with inclusion of smoking status as a covariate. When applied, feature selection was performed using Elastic Net regularization of R-glmnet, with the optimal alpha (mixing parameter) selected by cross-validation across values from 0.1 to 1. Stable features were defined as those consistently selected across 50 subsamples, each stratified by outcome and smoking status. Optimal modeling conditions were selected based on outer test set AUC performance, and seeds used for blind testing corresponded to the 50th or 75th percentile of AUCs observed across training repetitions.

**Blind testing:** For blind testing, 80 serum samples were selected by Vanderbilt University to match the samples used for model development with two additional criteria: The selection only included samples of high quality (those with an estimated hemolysis less than 50 mg/dL and storage times of 4 years or less) and samples from patients who were current and former smokers. The sample size of 80 was selected to have power to identify significant models with AUC of ROC ≥0.62 using an established method [33]. Patient status and clinical data were blind to researchers at time of prediction. However, information on approximate overall ratio of case to control samples, and nodule size and smoking status were available. Samples were shipped in one batch and processed in random order in 4 LC-iCAP batches and analyzed by NanoString Plexset on one plate. Due to lack of availability, a different code set lot was used for Plexset processing than was used for the training samples. By analyzing the 31 DMOG controls included in LC-iCAP NanoString Plexset processing, we identified ineffective calibration of the two code set lots. To mitigate this, we identified and omitted genes with significant differential expression between DMOG controls of code set 1 (training samples) versus code set 2 (test samples) (p-value <0.005). 9 models were tested on the blind test with different miscalibration correction strategies, including 5 before and 4 after quality control-based gene omission.

**LC-iCAP models M3 and M4.** RF models M3 and M4 selected for blind testing used either all 35 Plexset genes not removed due to code set calibration failure, or a subset of 14 genes remaining after feature selection described in the modeling section. Both models used smoking status as a covariate in interaction terms but not as a stand-alone feature.

**Mayo Clinic Model.** Pre-test risk of cancer malignancy was calculated for the test set by researchers at Vanderbilt University using Solitary Pulmonary Nodule (SPN) Malignancy Risk Score (Mayo Clinic model) [34]. This model uses 6 clinical risk factors including nodule spiculation, upper lobe location, smoking status (current or former vs non), nodule diameter, age, extrathoracic cancer diagnosis ≥5 years prior. The test excludes patients with prior lung cancer diagnosis or with history of extrathoracic cancer diagnosed within 5 years of nodule presentation.

**Integrated LC-iCAP model.** Prototype integrated classifiers were developed after blind testing by integrating LC-iCAP model M3 or M4 with the Mayo Clinic model using an approach similar to that used for the Nodify XL2 [9]. This approach is a decision tree model whereby the Mayo model's output is conditionally adjusted by a fixed amount based on a threshold established using the liquid biopsy readout: For LC-iCAP probabilities below the threshold, the Mayo model's output was reduced by a fixed amount and for probabilities above the threshold, the Mayo model's output was used without adjustment. See Fig. S12 for the technical parameters.

The integrated model performance on the blind test set was compared to that of the Mayo model by generating ROC curves for both models and comparing fixed decision thresholds corresponding to maximum specificity at ≥ 90 % sensitivity and ≥95 % NPV (with 25 % disease prevalence) using exact binomial version of McNemar's test [35]. Model calibration was not required for this comparison because it used points from the ROC curves corresponding to specific performance metrics rather than the absolute probability estimates. For verification, both models were calibrated to the test set prevalence using logistic regression (R-caret), and identical ROC curves were regenerated.

**Control for error and bias.** Key biological resources were authenticated: an aliquot of the 16HBE indicator cells was validated midway through the study externally at IDEXX bioanalytics by CellCheck 16™ Plus, and it passed all three tests. The 16HBE cell line of origin was confirmed to be correct and no contamination from *mycoplasma spp.* or other species was detected. Serum samples were assessed for hemolysis and storage time and omitted if above established thresholds. Patient gender age and smoking history were approximately balanced between classes. All DNA constructs were sequenced. All chemical resources were from reputable commercial sources.

Controls in data generation included: blinding researchers to disease status; randomizing sample positions; balancing classes within batches; balancing patient attributes between batches; using moat plates to control edge effects in the cell-based assay; developing standard controls and using them to monitor for technical failures; performing reproducibility studies; measuring RNA integrity before RNA-seq and omitting samples below a threshold 7; and detecting and correcting biases from RNA-seq (GC bias and batch effect) and NanoString Plexset analyses (batch effect).

Modeling practices were used that control for biases and overfitting including: training sets were balanced with approximately equal numbers of case and control samples; all models contained fewer features than samples to prevent overfitting (except for the pilot model, which had 25 features and 12 samples); and model validation included testing on independent held-out samples to mitigate overestimation of model performance regardless of sample size [36].

For final model development and blind validation, the 85 features were pre-validated using a different gene expression detection platform (i.e. all features were from models with significant performance in both cross-validation and on an independent held-out samples using LC-iCAP RNAseq data), and samples for blind testing had temporal independence from the training set for both sample collection and processing. Test set sample size was selected to have power to detect significant performance of a model with AUC of ROC ≥ 0.62 [33].

## 3 Results

The iCAP is a multivalent platform for blood-based diagnostics that captures complex, disease signals from patient serum by using standardized cells as biosensors. Multivariate gene expression responses of the cells are then analyzed using machine learning to predict cancer status (Fig. 1A). To evaluate the utility of the iCAP for lung cancer screening, we optimized and validated the LC-iCAP for classification of patients with IPNs identified by CT scan and mesured the utility of the predictions for nodule management.

**Study Summary:** Serum samples were collected from patients with IPNs identified by CT scan and later characterized as malignant or benign based on diagnostic biopsy/resection or ≥2 years of serial imaging follow-up (Table 1).

Archived samples were used to develop the LC-iCAP in two stages: assay development and analytical validation, followed by early-stage clinical validation (Fig. 1B). The first stage addressed optimization of model parameters, assay reproducibility, analytical variation, and control standardization. It also included selection of 85 LC-iCAP gene expression features for final model development in the next stage. Classifier development during this stage used training and held-out validation sets to evaluate model performances and guide parameter and hyperparameter tuning. In the second stage, patient samples were analyzed using a high-throughput version of the LC-iCAP for final model development and validation using PRoBE design (prospective-specimen-collection and retrospective-blinded-evaluation) [37]. It used nested cross-validation for model training, followed by blind testing on a temporally and procedurally independent test set to assess the performance of fully specified models.

In all modeling studies, sample sizes were guided by power calculations, and sample exclusions were made before validation. Validation sets were kept fully independent from tuning sets, and for blind validation, the research team remained blinded to test set classes until all predictions were finalized. To minimize overfitting, models were constrained to use fewer features than samples [38,39]. Additionally, feature selection and final model testing were performed on different gene expression platforms (RNA-seq and NanoString PlexSet, respectively) to reduce analytical bias. This two-step modeling approach of locking the features prior to final model training follows practices established in development of validated multivariate diagnostic assays such as PAM50 and Oncotype DX to ensure unbiased assessment of classifier generalizability [11,12]. Detailed methods follow below.

### 3.1 LC-iCAP development and analytical validation

**Proof of Concept:** To establish proof of concept for the LC-iCAP assay, we trained and tested an initial classification model. We first acquired Cohort 1 from Vanderbilt University consisting of serum samples from patients with IPNs, including six with lung cancer and six with benign nodules (Table 1, Data File 1). Samples were processed in the LC-iCAP assay alongside reference serum controls. RNA-seq analysis identified 239 differentially expressed genes (DEGs) between cases and controls (FDR <0.05, Benjamini-Hochberg) and functional enrichment analysis using STRING revealed that these DEGs formed a significantly interconnected network with enrichment for hypoxia-inducible factor 1-alpha (HIF1A) signaling (KEGG) and response to hypoxia (Gene Ontology), both of which are biologically relevant to lung cancer [40]. We next trained a Random Forest (RF) classifier on Cohort 1 data using the top 25 DEGs ranked by FDR as features with 10 random seeds and internal cross-validation to generate a pilot LC-iCAP model for lung cancer prediction.

We tested the pilot model on LC-iCAP data from an independent set of 103 serum samples (Cohort 2; Table 1, Data File 1). The classifier demonstrated significant performance with a median AUC of 0.62 (95 % CI: 0.51–0.73; Fig. 2A). Similar performance was observed when models were trained using the top 50, 75, or 100 DEGs (data not shown).

To further evaluate signal generalizability, hierarchical clustering was performed on Cohorts 1 and 2 using the top 20 DEGs from Cohort 1. This approach broadly separated samples into two class-associated clusters (Fig. 2B), supporting the presence of lung cancer-specific signals in the readout.

Together, these data establish feasibility of the LC-iCAP approach for lung cancer detection. However, the results also suggest potential sources of variability in the readout, including patient heterogeneity, serum quality, and analytical factors inherent to the assay or RNA-seq. These sources of noise were investigated further during assay optimization.

**Assay Controls and Standards:** Biological and chemical standardized controls were developed to support assay optimization, monitor performance, and assess reproducibility.

**Fig. 3:**
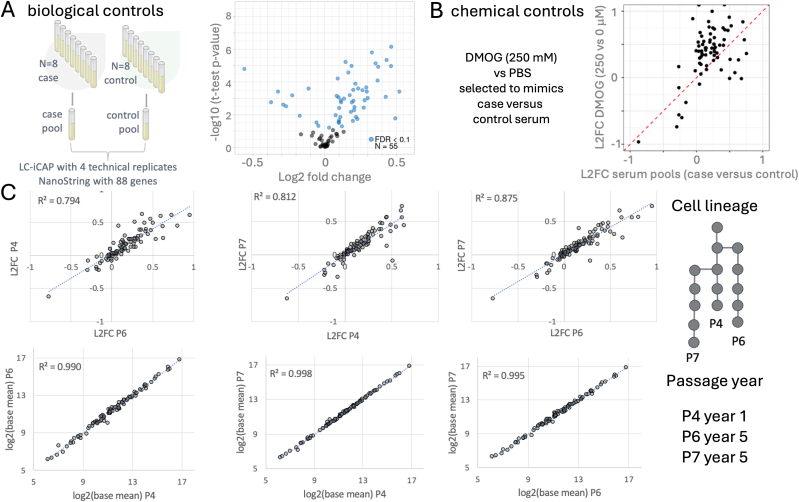
Development of assay controls and demonstration of stability of LC-iCAP indicator cells. A. Biological controls were pools of case and control patient serum samples. The volcano plot shows the quantitative assay readout for these controls - differential expression of ∼55 of 88 genes measured by NanoString. B. Chemical controls were DMOG versus PBS, which were selected to mimic the HIF1A/hypoxia response to case versus control sera of cohort 1. The scatter plot shows that the LC-iCAP-NanoString readout using the chemical and biological controls is broadly coherent. C. LC-iCAP differential expression is reproducible across 3 different passages of indicator cell generated over 4 years (P4, P6, and P7). LC-iCAP NanoString data were generated for 4 replicates of case and control serum pools across the three different passages of indicator cells. Graphs show pairwise comparisons of expression levels (lower) and differential expression levels (upper). The R² values of the linear models indicate that the three cell passages exhibit similar variability, supporting the stability of the cell line for clinical assay development. Other metrics of comparison are shown in Figure S2A. L2FC, log2 fold change.

Biological standards consisted of pooled case and control sera, each generated by combining aliquots from eight individuals in Cohorts 1 and 2 (indicated in Fig. 2B). These serum pools were assayed using the LC-iCAP with a targeted NanoString panel comprising 58 DEGs from Cohort 1, along with 30 additional genes (Fig. S2; Data File 2). NanoString analysis of four technical replicates of each pool identified 55 genes as differentially expressed between case and control samples (FDR <0.1) (Fig. 3A), establishing a baseline biological signal for use in subsequent optimization studies.

Chemical standards included a phosphate-buffered saline (PBS) control versus DMOG, a small-molecule agonist of the hypoxia pathway, selected to mimic the malignant serum-induced hypoxia response observed in Cohort 1. In an initial experiment (see Methods), DMOG exposure in indicator cells triggered differential expression of 24 of the 58 development genes. A scatter plot of log-fold changes for both standards demonstrates strong concordance between the DMOG and serum-induced responses across the NanoString panel (Fig. 3B), supporting the use of both biological and chemical controls to benchmark assay behavior.

**Optimization of Assay Parameters:** Assay parameters were optimized with the goal of detecting and controlling for unwanted variation from analytical sources to improve stability and magnitude of differential expression and thus model performance. To do this, we assayed 4 technical replicates of case and control serum pools in the LC-iCAP with the NanoString readout under various assay parameters and optimal conditions were selected that maximized the number of significant DEGs and/or the separation of the classes by principal component analysis (PCA). We tested 4 candidate indicator cell types (16HBE, A549, MRC5, and Nuli-1); 2 serum incubation times (6 h, 24 h); 4 serum concentrations (1 %, 5 %, 10 %, and 20 %); and the effect of Trichostatin A (TCA) addition, an inhibitor of the hypoxic response. The optimal LC-iCAP parameters were found to be a 24 h incubation of either 5 % or 10 % serum with 16HBE lung epithelial indicator cells in the absence of TCA, which matched the baseline conditions used in proof of concept (Fig. S1). To avoid potential bias from measuring only a subset of genes, RNA samples from the TCA and cell type experiments were reanalyzed by RNA-seq, which yielded similar results (data not shown).

In addition to the analytical optimizations of the cell-based assay listed above, we detected and corrected sources of noise arising from LC-iCAP and RNA-seq batch effects, and artifactual duplicate reads and GC biases in the RNA-seq data. All corrections improved model performance and increased the detected number of DEGs across classes (data not shown), and thus were implemented in data processing going forward.

**Analytical Reproducibility Testing:** We next evaluated the analytical reproducibility of the LC-iCAP by testing gene expression stability across multiple experimental conditions. To address the long-term stability of the cells, we tested the serum pool standards in the LC-iCAP using three different lots of indicator cells derived from distinct lineages, expanded over a four-year span. Pair-wise comparisons of gene expression between lots showed strong linear correlations (R^2^ > 0.99; Fig. 3C). Comparisons of differential expression also showed good agreement (R^2^ > 0.79; Fig. 3C) and PCA plots demonstrated consistent separation of case versus control samples across all lots (Fig. S2A). These results support the long-term stability of the cells for clinical application.

Next, batch-to-batch and platform reproducibility were tested. We tested the pooled serum standards across different LC-iCAP batches run on different days, different gene expression detection platforms (RNA-seq versus NanoString) and different NanoString batches. Gene expression profiles remained highly correlated across all conditions (R^2^ > 0.99) and differential expression levels also showed strong reproducibility (R^2^ = 0.79–0.97) (Fig. 3C–S2A-D). We also compared expression profiles across conditions using technical replicates of individual serum samples, showing that 92–98 % of genes were significantly correlated between conditions (FDR <0.1; Fig. S2B–D).

In these experiments, we assessed both gene-level variability (differential expression) and sample-level variability (rank consistency), with the former being a more stringent metric that is better suited for assessing signal to noise for subtle biomarker signatues [41]. These data showed that the LC-iCAP has strong analytical reproducibility and can detect signal above background across various conditions used in assay development. Therefore, the variability seen in the earlier proof-of-concept experiment (Fig. 2B) likely arises from pre-analytical sources, not analytical instability.

**Fig. 4:**
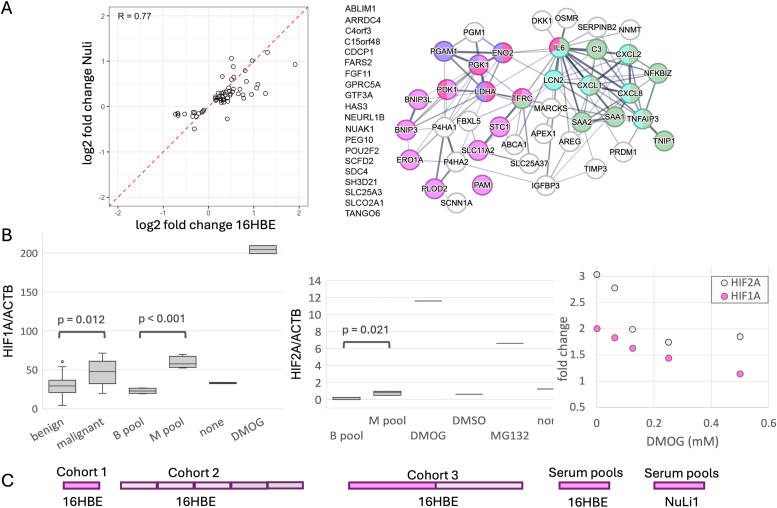
The LC-iCAP response to case versus control serum is enriched for HIF1A-mediated hypoxia signaling in multiple bronchial epithelial cell lines. A. Left, comparison of case versus control differential expression in LC-iCAP RNAseq data using two different bronchial epithelial indicator cell lines (16HBE and NuLi-1), each with 4 replicates of pooled serum controls. The 61 genes with differential expression in both cell types are shown (FDR <0.1). Right, STRING network analysis of proteins encoded by the upregulated genes shows significant enrichment of interactions (p-value <0.001) and functional annotation clusters, including HIF1A/response to hypoxia (red/pink), glycolysis (purple), and IL17 signaling/inflammation (cyan/green) (FDR<0.001). Edge thickness represents confidence of interactions. Proteins without known interactions or annotations are listed at the left. B. Results of Western blot analysis showing upregulation of HIF1A and HIF2A transcription factors in the LCiCAP with 16HBE cells in response to case versus control serum. Left and middle, box plots showing levels of HIF1A or HIF2A normalized to actin across 4 replicates of pooled serum controls (M pool and B pool) and across 28 different individual serum samples including 14 of each class (malignant and benign). The 28 Individual samples included the 12 samples used to make the pools. Positive controls were one or two replicates each of DMOG or Mg132 versus DMSO or no stimulus. Right, one replicate of each serum pool was analyzed in the LC-iCAP with increasing concentrations of DMOG showing that DMOG dampens case versus control differential expression for both factors. Western blot images are in Figure S5. C. Case versus control differential expression and enrichment of ‘HIF1A signaling’ and ‘response to hypoxia’ were observed in various LCiCAP RNAseq experimental batches in this study (FDR <0.01) (pink boxes), whereas others had very weak or no detectable differential expression (gray boxes). All LC-iCAP models with significant performance in this study used features selected from one or both of the hypoxia-enriched batches of cohort 1 or 3.

**Validation of Hypoxia Signaling as a Generalizable LC-iCAP readout**: To evaluate the generalizability of the hypoxia response identified in Cohort 1, we analyzed the LC-iCAP readout using the pooled serum standards, composed of 16 individual samples, 12 of which were distinct from those in the original Cohort 1 (Fig. 2B, bottom). First, RNA-seq data from two pooled serum control experiments (n = 8; Datafile 2) were combined and analyzed for differential expression between case versus control groups. GSEA identified hypoxia as the most enriched pathway (adjusted p-value <0.05, Fig. S4). Next, we repeated the LC-iCAP with the pooled serum standards using a different normal bronchial epithelial cell line (NuLi1) as indicator cells. We identified 61 genes that were significantly differentially expressed in both LC-iCAP RNA-seq experiments (using 16HBE and NuLi1 cells), with expression changes showing strong correlation between cell types (R 0.77, Fig. 4A, *left*). STRING network analysis of the 47 coherently up-regulated genes revealed high network connectivity and enrichment of HIF1A/response to hypoxia and other processes (FDR <0.001, Fig. 4A, *right*).

**Fig. 5:**
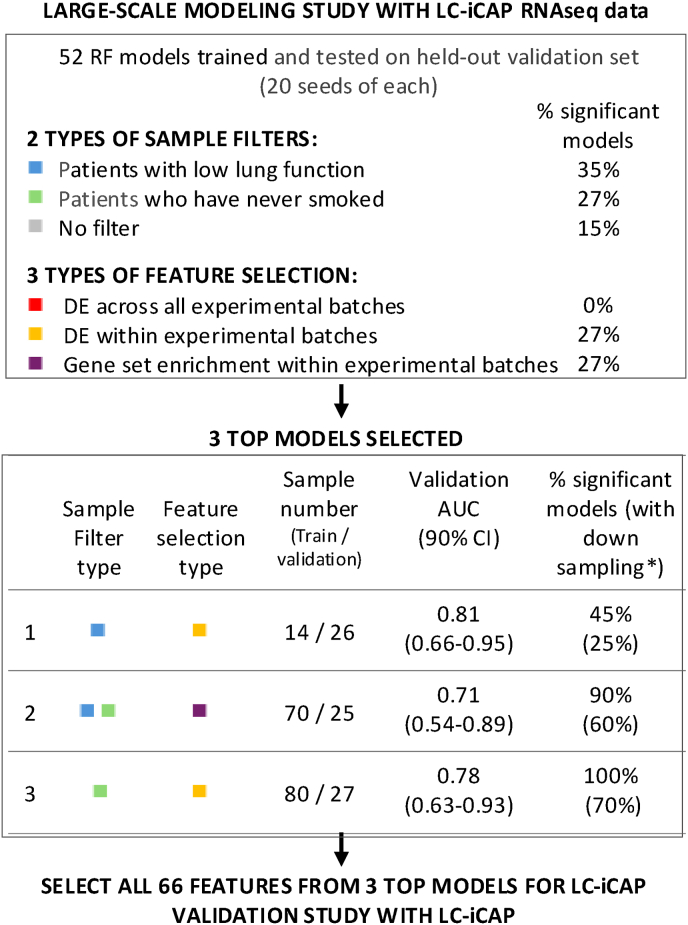
Summary of a large-scale modeling study with LC-iCAP RNA-seq data to identify a subset of optimal features for final model development with LC-iCAP NanoString Plexset data. First, 52 RF models were trained using various parameters, each with 20 seeds, and tested on a held-out validation set. Results are summarized (top). Next, three top performing models were chosen (bottom) and the 66 features from these models were selected for the validation study. % significant models is the percentage of models in the category having at least one seed with significant performance. ∗model training was performed with and without down sampling to balance classes within each experimental batch. DE, differential expression.

Towards elucidating the response mechanism, we compared levels of hypoxia-responsive transcription factors HIF1A and HIF2A between case and control classes in the LC-iCAP using quantitative western blotting on both serum pools and individual samples. Significantly higher levels of both factors were observed with case versus control sera, an effect reduced by the addition of DMOG, a known HIF1A stabilizer (Fig. 4B). Collectively, these results support that hypoxia signaling is a generalizable marker of lung cancer in the LC-iCAP and suggest the involvement of HIF1A and HIF2A in the response observed in the indicator cells.

Throughout assay development, several LC-iCAP experimental batches had enrichment of ‘HIF1A signaling’ and ‘response to hypoxia’ GO molecular processes in case versus control differentially expressed genes (Fig. 4C*,* Data file 2), whereas batches without enrichment had very few if any differentially expressed genes with FDR <0.1.

**Selection of model features for assay validation:** Although LC-iCAP RNA-seq data is well-suited for assay optimization and feature discovery, it lacks the scalability required for clinical deployment. To enable a high-throughput (HTP) implementation, we used RNA-seq data to predefine a fixed set of 85 features for final model training and validation using NanoString PlexSet, a HTP platform for analysis of up to 96 genes across 96 samples per batch. This cross-platform strategy helps mitigate platform-specific biases and enhances the robustness and generalizability of the final model. Importantly, locking down a prespecified feature set prior to final model training and blind testing is a rigorous approach aligned with established practices used in the development of clinically validated gene expression classifiers, such as PAM50 and Oncotype Dx [11,12].

To select the features, we used an unsupervised modeling-based approach. We conducted a large-scale model parameterization study with LC-iCAP RNA-seq data and then selected features from 3 top models with optimal parameters (outlined in Fig. 5). We used data from cohorts 1–3 (Table 1), filtered samples based on sample and data quality (see Methods) and partitioned them into a training and held-out validation set. Next, we trained 52 RF models with 20 seeds each, tested them on the validation set and ranked model parameters based on performance. Feature selection was based on case versus control differential expression either across all samples or within sample subsets, an approach taken to improve signal detection in the presence pre-analytical variability in the data. Optional sample filters to remove either non-smokers or patients with low lung function were applied based on our findings (Fig. S6) or those of other studies [42]. Notably, the highest-performing models consistently applied one or both sample filters and favored features identified from sample subsets with strong differential expression rather than those derived from the full training set, suggesting the importance of accounting for clinical heterogeneity in model development.

Three top models were selected with high performance, including one with AUC of 0.78 (90 % CI 0.63–0.93) with 100 % sensitivity and 60 % specificity (Fig. 5 bottom). A list of 85 features was generated for the LC-iCAP validation study composed of 66 features selected from multiple seeds of the 3 top models and an additional 19 genes from the proof-of-concept pilot model not yet in the list (Data file 3).

### 3.2 Early-stage clinical validation

To validate the LC-iCAP, we generated LC-iCAP NanoString PlexSet data for the fixed panel of 85 pre-selected gene expression features and used these data for final model development and blind testing. In this study, we trained models using nested cross-validation across cohorts 1–3, then selected fully parameterized models for testing on a blind test set of 79 samples, which were temporally and procedurally independent from the training set. This validation approach was designed to eliminate potential expression platform-specific biases or biases in sample collection and processing. Additionally, the fixed 85-gene panel serves as a constrained search space for training and validating classifiers with new sample sets in the future, such as optimized versions of the nodule risk classifier, or multiclass models that distinguish benign nodules from tumors with low or high risk of malignancy, as previously demonstrated in breast cancer molecular subtyping [12].

**Fig. 6:**
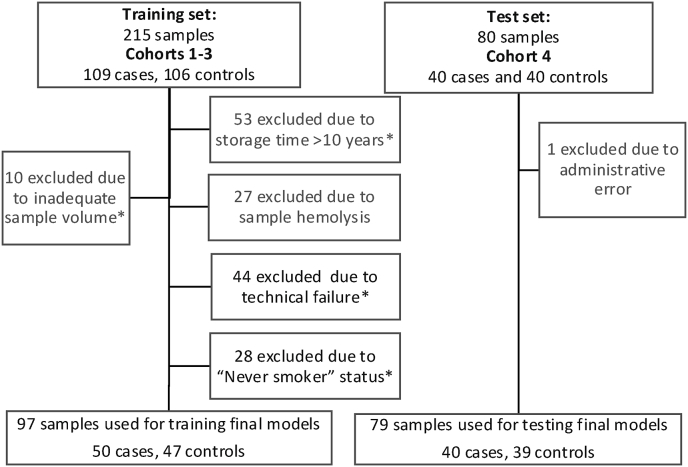
Retrospective sample flow diagram for LC-iCAP validation study. Cohorts 1–3 were used for model training and cohort 4 was used as a blind test set. The training set and blind test set had temporal independence; test samples were collected 9 years later and assayed 1 year later than the training samples. Sample numbers are not cumulative as samples can belong to multiple exclusion groups. ∗Samples indicated were used for LC-iCAP development and/or feature selection.

**Generating LC-iCAP data with high-throughput readout:** We generated LC-iCAP data using the NanoString PlexSet platform for model training across cohorts 1–3, including some samples previously used for feature selection (Table 1, Data File 4). After merging and normalizing the data, we applied filtering based on sample and technical quality, and patient characteristics, resulting in a final dataset of 97 samples for modeling (Fig. 6). This included: 1) Excluding data from the small number of ‘never smokers’ based on the results of assay optimization, which also improved the relevance of this study to lung cancer screening, which is for current or former smokers only [6], 2) Removing data from samples with hemolysis using an established threshold [17], which was also implemented in assay optimization, and 3) Excluding low-quality samples with long storage times based on our analysis showing storage times >10 years affect the LC-iCAP readout (Figs. S7 and S8), which is consistent with known effects of storage time on blood biomarkers [43,44].

After data generation, we tested LC-iCAP Plexset reproducibility by hierarchical clustering using data from the standard control samples and found reproducibility of expression and differential expression across the 5 Plexset plates (Fig. S9, code set lot 1 samples).

**Final LC-iCAP model training:** We used the Plexset data for the 97 samples as a training set for model development. Feature selection was performed from the fixed panel of 85 genes and 8 housekeeping genes, which were included to provide a stable baseline which can enhance model robustness as shown previously [45]. Model parameters and hyperparameters were selected using nested cross-validation, a recommended approach for small datasets to prevent information leakage and provide unbiased estimates of model performance [36]. Most parameters used were informed by prior assay optimization (see Fig. 5), with the addition of patient smoking status (current or former) as a covariate. Top-performing models were selected for blind validation based on outer test set AUC.

**Final blind testing of LC-iCAP models:** For blind testing of locked-down models, a new set of 80-samples from Vanderbilt University was added to the study (Cohort 4, Table 1, Table 2). Samples were from patients with IPNs with a pretest risk of malignancy between 5 and 65 % calculated using the Mayo Clinic model. The patient characteristics matched those of the training set, except and all had high serum quality with storage times between 1 and 4 years. Additionally, samples of the blind set had temporal and processing independence from the training set, with a collection window that was 9 years later and LC-iCAP processing that was 1 year later, adding more rigor to the validation than concurrent collection and processing [46].

**Table 2:** Participant demographics for LC-iCAP validation study.

**Characteristic**	Train, n = 97				Test, n = 79			
	**N**	**Control**^c^, n = 47	**Case**^d^, n = 50	**p-value**	**N**	**Control**^c^, n = 39	**Case**^d^, n = 40	**p-value**
Age, Mean (SD)	97	63 (9)	64 (8)	0.50^a^	79	64 (8)	65 (7)	0.72^a^
sex, n (%)	97			0.59^b^	79			0.24^b^
Female	38	19 (40)	19 (38)		28	12	16	
Male	59	28 (60)	31 (62)		51	27	24	
smoking status, n (%)	97			0.02^b^	79			0.08^b^
current	43	25 (53)	18 (36)		35	20	15	
former	54	22 (47)	32 (64)		44	19	25	
pack years	97	53 (28)	54 (34)	0.90^a^	79	60 (35)	62 (28)	0.741
nodule size, Mean (SD)	97	12.24 (5.97)	17.82 (4.35)	<0.001^a^	79	12.07 (5.33)	17.87 (6.53)	<0.001^a^
storage years, Mean (SD)	97	8.10 (1.59)	6.49 (2.18)	<0.001^a^	79	3.44 (0.72)	3.65 (0.62)	0.16

a Welch Two Sample *t*-test.

b Person's Chi-squared test.

c Samples from patients with benign IPNs.

d Samples from patients with malignant IPNs. IPN, indeterminate pulmonary nodule.

**Fig. 7:**
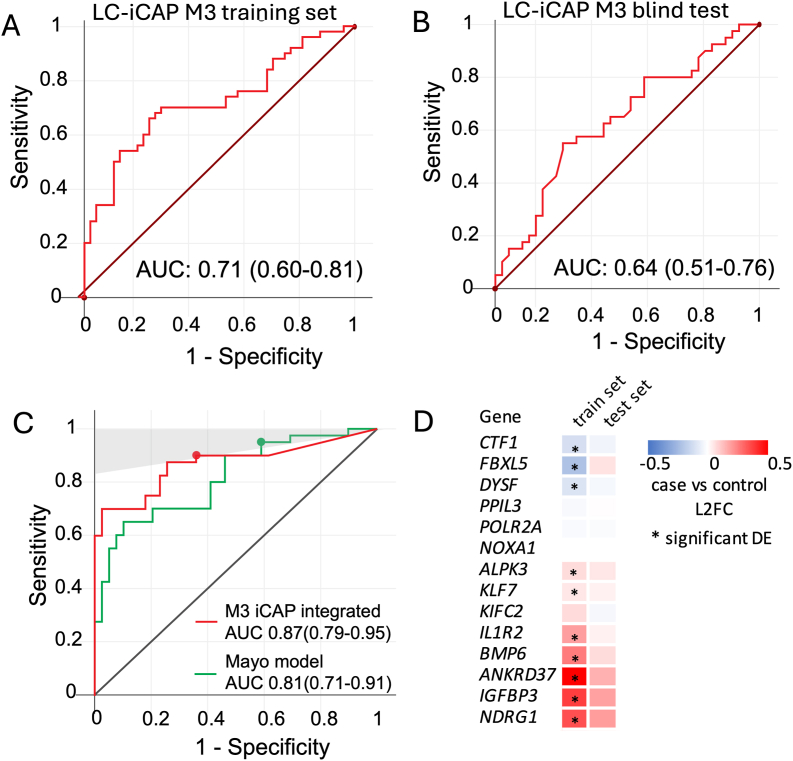
ROC curves showing performance of the M3 LC-iCAP model and the M3 iCAP-integrated classifier in temporal blind validation on the test set. A and B The M3 LC-iCAP model demonstrated significant performance based on out-of-bag (OOB) predictions from the training set (A) and on an independent blind test set (B) C. The iCAP-integrated classifier outperforms the Mayo Clinic model on the test set. The shaded region corresponds to ≥95 % NPV using a 25 % prevalence estimated in the clinical population. The model performances were compared at cut points with clinical utility as ruleout tests (with ≥95 % NPV and ≥90 % sensitivity) (colored nodes) (corresponding to 96.1 % NPV, 41 % specificity and 95 % sensitivity for the Mayo model and 95.1 % NPV, 64 % specificity and 90 % sensitivity for the iCAP integrated classifier). The integrated classifier had significantly higher specificity than the Mayo model (exact binomial test p-value = 0.049) at the cut point. AUCs are shown with 95 % confidence intervals in brackets. D. Heatmap of LC-iCAP NanoString Plexset data showing case versus control differential expression for genes of model M3. For samples of the training and test sets, median differential expression values are shown. Genes with significant differential expression are marked with asterisks (Mann Whitney *U* test p-values <0.05). Training versus test set differential expession profiles had a strong pearson correlation coefficient of 0.8 (p-values 5.4E-4) showing good reproductivity of expression between traning and blind test sets. L2FC, log2 fold change.

The blind samples were analyzed by LC-iCAP-NanoString Plexset and data quality was checked by analysis of 31 DMOG chemical controls run with code set lot 1(training) and code set lot 2 and (blind test set samples). This identified a miscalibration between the training set and test sets that measurably affected 50 of the 85 genes, which was attributed to technical failure of normalization between the code set manufacture batches (Fig. S9, Data file 4). To address this, the control samples were used to devise three approaches for mitigating the miscalibration, which were evaluated by testing nine models on the blind test set. Of these, both random forest (RF) models that omitted the 50 miscalibrated genes (M3 and M4) demonstrated discriminative performance, with AUC confidence intervals exceeding 0.5 (Fig. S10, Fig. 7B, Fig. S11B).

After blind testing was complete, gene features of both models were analyzed for case versus control differential expression in the training and blind test sets. Comparison of training and test set profiles showed significant correlation for both M3 (R = 0.8, Fig. 7D) and M4 (R = 0.73 Fig. S11D). This supports the biological validity and generalizability of the gene signatures used by the models. However, no genes had significant differential expression in both sample sets suggesting patient-to-patient variation and the importance of using a multivariate signature.

**Exploring clinical implementation of the LC-iCAP:** To explore the potential clinical utility of the LC-iCAP, a prototype clinical version of the assay was developed after blind testing by integrating the final LC-iCAP model with the Mayo Clinic model [34] using an approach similar to that used for the Nodify XL2 test offered by Biodesix [9] as described in Fig. S12. Performance of this ‘iCAP integrated classifier’ was compared to that of the Mayo Clinic model at specific cut points on the ROC curves with clinical utility to rule-out cancer (corresponding to sensitivity ≥90 % and NPV ≥95 % using an estimated cancer prevalence of 25 % in a community pulmonary practice) [13,14]. At this threshold, the specificity of the iCAP integrated classifier was significantly better than that of the Mayo Clinic model suggesting clinical utility of the LC-iCAP (McNemar exact binomial test p-value [35] = 0.049) (Fig. 7C). Similar results were observed with the other validated LC-iCAP model (Fig. S11C). Because the models were compared at ROC curve points with specific performance metrics (rather than absolute probability estimates), model calibration was not required and when implemented had no effect on the results (data not shown). The parameters of the integrated classifier were selected using the blind samples; while the study demonstrates the potential performance of the LC-iCAP model, the fixed constants of the integrated classifier remain to be independently validated.

## 4 Discussion

The iCAP is a biosensor assay designed to overcome the signal-to-noise limitations of blood-based diagnostics by capitalizing on the evolved ability of cells to detect and respond to weak signals in noisy environments (Fig. 1A). The iCAP transforms multivalent disease signals in blood into a standardized gene expression readout from cells, enabling applying well-established tools from cell biology to the emerging field of blood-based diagnostics.

We developed the LC-iCAP, a multivariate blood test to improve risk assessment in patients with IPNs detected by CT screening, aiming to reduce unnecessary biopsies for benign cases while focusing further evaluation on malignancies. The cell-based approach enabled enrichment analysis of the case–control differential readout, revealing a hypoxia-related signature, and biochemical analyses implicating HIF1A and HIF2A as key transcriptional regulators (Fig. 4). All classifiers with significant performance included hypoxia-related genes drawn from experimental batches with differential hypoxia enrichment (Fig. 4C). These findings are consistent with the established role of hypoxia in lung and other cancers [40,47,48], and suggest that the LC-iCAP readout reflects tumor-associated blood analytes.

The cell-based platform enabled development of standardized biological and chemical controls with quantitative, multicomponent readouts. These were critical for assay optimization (Fig. S1) and helped identify and exclude two technical failures (Figs. S3 and S9), underscoring their value as built-in safeguards for assay integrity.

We also used these controls for analytical validation, demonstrating reproducibility of the case–control differential expression across multiple conditions (Figs. S2, 3C, and S9). This included a consistent readout from the 16HBE indicator cells across lineages, supported by their extensive use over 35 years [49], and generalizability of the differential hypoxia response across distinct bronchial epithelial cell types (16HBE and NuLi1) (Fig. 4A). These findings support the robustness and stability of the LC-iCAP readout for clinical deployment and highlight the value of these controls for ongoing monitoring during future assay deployment.

Despite strong analytical reproducibility, we observed noise in the assay readout: the hypoxia-related differential expression that underpinned disease classification was variable across sample subsets and effect sizes were small (Fig. 4, Fig. 7D, and S11D). This variability is likely due pre-analytical factors like those we have characterized in this study including variation in lung function and serum storage duration across samples (Fig. S6–S8). These findings underscore the importance of controlling for pre-analytical variation and using ensemble modeling approaches like RF in liquid biopsy development studies.

As an early-stage clinical validation of the LC-iCAP, we developed and validated predictive models for lung nodule management in two stages. In stage 1, we used a modeling-based approach to reduce the transcriptomic feature space from ∼20,000 genes to a fixed panel of 85 candidates. These genes were selected based on their occurrence across four LC-iCAP RNA-seq models tested on independent validation sets: one pilot model trained on 12 samples and validated on an independent set of 103 samples (AUC = 0.62; Fig. 2A), and three optimized models showing stronger performance (AUCs = 0.71–0.91; Fig. 5). These 85 genes and 8 housekeeping genes were locked down for clinical assay development and validation, a strategy that is consistent with standard practices in clinical gene expression classifiers such as PAM50 and Oncotype DX [11,12]. This approach serves multiple purposes: it minimizes the risk of overfitting in this and future clinical validation studies by constraining the search space, it aligns with regulatory expectations from agencies such as the FDA, and it enables the development of high-throughput, clinically deployable assay configurations.

In stage 2, the fixed gene panel was used to train and validate a high-throughput version of the assay using NanoString PlexSet. Model training was done by nested cross-validation on samples from current and former smokers with IPNs using the fixed gene set as features with a smoking status covariate (Table II). Final blind testing was performed on a 79-sample test set from patients with IPNs, including 39 with benign nodules and 40 with malignant nodules (72 % Stage I, 22 % Stage II). These samples were collected and processed independently of the training set, enhancing the rigor of validation compared to randomly partitioned dataset [46].

We then assessed the predictive performance of these models on the independent, blinded test set. Two final models achieved AUCs of 0.64 (95 % CI: 0.51–0.76) and 0.62 (95 % CI: 0.50–0.75), suggesting performance exceeding random expectation (Fig. 7C and S11C). The generation of multiple performant models in this study using overlapping gene subsets highlights the coregulatory structure of gene expression networks and underscores the potential of cell-based transcriptional profiling to capture biological heterogeneity relevant to diagnosis. Both validated models included genes previously linked to hypoxia and HIF1A signaling, such as *Ankyrin Repeat Domain 37* (*ANKRD37*), *insulin Like Growth Factor Binding Protein 3* (*IGFBP3*), and *N-Myc Downstream Regulated Gene* 1 (*NDRG1*), as well as *Bone Morphogenetic Protein 6* (*BMP6*), encoding a transcription factor known to induce *HIF1A* under hypoxic conditions [50]. Supporting generalizability of the gene set, post-hoc analysis showed case-versus-control differential expression patterns for classifier genes were highly concordant between training and blinded test sets (Fig. 7D and S11D).

After blind testing, we further explored the clinical implications of the final LC-iCAP model M3 recognizing it as one example of the validated gene set's predictive potential. We did this by integrating it with the Mayo Clinic model using a method previously developed for deploying the Nodify XL2 test by Biodesix [9] (Fig. S12). The ‘iCAP integrated classifier’ was evaluated against the Mayo model at cut points yielding ≥95 % NPV, assuming a 25 % disease prevalence in the intended-use population [13,14]. The threshold was selected based on the ROC curve from the blind test set to reflect performance in a clinically meaningful rule-out setting. At this threshold, the integrated classifier achieved 64 % specificity and 90 % sensitivity, significantly outperforming the Mayo model and supporting its potential clinical utility (Fig. 7C).

In clinical use, specificity at the rule-out threshold reflects the proportion of patients with benign nodules who could be safely directed to surveillance, avoiding unnecessary invasive procedures. At 64 % specificity, the LC-iCAP integrated classifier outperformed existing CT-integrated blood tests from Biodesix and MagArray, which report specificities of 44 % and 33 %, respectively, representing a 1.45–1.9-fold improvement [8,9]. This indicates a higher proportion of actionable results for patients with benign nodules. The classifier's sensitivity was 90 %, slightly lower than the 97 % and 94 % reported by the other tests, but it still achieves 95 % NPV in the intended-use population. This reflects a better balance between avoiding harm and improving utility.

While the data are promising, this study has limitations. The study used archived samples with variable storage durations, and there was a covariate shift between training and test cohorts (median 7.6 years vs. 0–4 years, respectively), which may have negatively impacted model performance. In addition, to minimize a NanoString Plexset-specific technical bias, we evaluated nine classifier variants during blind validation, using parameters informed by analysis of the chemical control samples included in the assay. We anticipate that future clinical validation studies conducted on diagnostic-grade gene expression platforms using the same fixed gene panel, such as NanoString nCounter Dx or Ion AmpliSeq, will enhance assay performance and reproducibility. As a next step, we plan to conduct a multi-site, prospective study with a larger and more balanced sample set to enhance model robustness, followed by a formal clinical utility study in alignment with recommendations from the American Thoracic Society [7].

The iCAP is a scalable, low-cost, multivalent platform with a multivariate readout, and may have future utility as a next-generation framework for cancer screening, including multi-cancer early detection (MCED). This could involve either using an array of indicator cells or tuning the LC-iCAP to detect multiple cancer types. Supporting this possibility, the LC-iCAP readout includes robust activation of the HIF1A-mediated hypoxia response pathway, a hallmark of tumor biology [47,48]. Notably, hypoxic tumors are more likely to metastasize and are less likely to respond to treatment [48] and to our knowledge, blood biomarkers of tumor hypoxia have not yet been well established. Future studies include exploring the use of fluorescent reporters and single cell analysis to further simplify and amplify the LC-iCAP readout.

To achieve the Cancer Moonshot initiative's goal of accurate early detection with minimal overdiagnosis and missed cases, relying on only one test is unlikely to yield optimal clinical utility. Multimodal approaches, which combine multiple orthogonal tests can outperform individual tests in isolation [35,51]. The iCAP is a non-conventional approach that is complementary to other more traditional tests and thus is well-suited for combinatorial diagnostics.

## Author contribution

JDB, FJD and MDD contributed equally to this work. JDA, JJS, and RJL and conceived the study, JDB and JJS designed the experiments. AV, PM, and SD selected and provided samples and insights related to clinical need. JDB, LRM and YQ conducted the experimental work and collected the data. Data analysis was performed by FJD, GAW, JDB, JJS, MDD, SAD, SD and YQ. The manuscript was written by JJS. All authors reviewed and approved the final manuscript. JDA, JDB, JJS and RJL supervised the project and provided critical insights throughout the study.

## Ethics approval and consent to participate

This study was approved by WCG Institutional Review Board (study 1283522). Banked specimens and clinical data used in this study were from subjects enrolled in the following previously IRB-approved studies: “Molecular Predictors of Lung Cancer behavior,” (NCT00898313, Vanderbilt University), “Gene-Environment Interactions in Lung Cancer” (IRB 806390, University of Pennsylvania), and “A Case Control Study of Smokers and Non-Smokers” (IRB 800924, University of Pennsylvania). All participants provided written informed consent prior to participation, and the study was conducted in accordance with the Declaration of Helsinki.

## Declaration of competing interest

The authors declare the following financial interests/personal relationships which may be considered as potential competing interests: Jennifer J. Smith reports financial support was provided by 10.13039/100000002National Institutes of Health 10.13039/100000054National Cancer Institute. Jennifer J. Smith reports financial support was provided by 10.13039/100000002National Institutes of Health National Institute of Aging. Anil Vachani reports financial support was provided by 10.13039/100000066National Institute of Environmental Health Sciences. Stephen Deppen reports financial support was provided by 10.13039/100000002National Institutes of Health 10.13039/100000054National Cancer Institute. Jason D. Berndt reports a relationship with PreCyte, Inc. that includes: consulting or advisory, employment, and equity or stocks. Mark D. DAscenzo reports a relationship with PreCyte, Inc. that includes: consulting or advisory, employment, and equity or stocks. Fergal J. Duffy reports a relationship with Seattle Children's Research Institute that includes: employment and a relationship with PreCyte, Inc. that includes: consulting or advisory. Leslie R. Miller reports a relationship with Seattle Children's Research Institute that includes: employment and a relationship with PreCyte, Inc. that includes: equity or stocks. Yijun Qi reports a relationship with PreCyte, Inc. that includes: consulting or advisory, employment, and equity or stocks. G. Adam Whitney reports a relationship with PreCyte, Inc. that includes: consulting or advisory, employment, and equity or stocks. Samuel A. Danziger reports a relationship with Seattle Children's Research Institute that includes: employment and a relationship with PreCyte, Inc. that includes: equity or stocks. Robert J. Lipshutz reports a relationship with PreCyte, Inc. that includes: board membership, employment, consulting or advisory, and equity or stocks. John D. Aitchison reports a relationship with Seattle Children's Research Institute that includes employment and a relationship with PreCyte, Inc. that includes: board membership and equity or stocks. Jennifer J. Smith reports a relationship with PreCyte, Inc. that includes: consulting or advisory, employment, equity or stocks, funding grants, and travel reimbursement. Jennifer J. Smith has patent pending to Seattle Children's Research Institute and PreCyte, Inc. Jason D. Berndt has patent pending to Seattle Children's Research Institute and PreCyte, Inc. Fergal J. Duffy has patent pending to Seattle Children's Research Institute and PreCyte, Inc. Mark D. DAscenzo has patent pending to Seattle Children's Research Institute and PreCyte, Inc. G. Adam Whitney has patent pending to Seattle Children's Research Institute and PreCyte, Inc. John D. Aitchison has patent pending to Seattle Children's Research Institute and PreCyte, Inc. Robert J. Lipshutz has patent pending to Seattle Children's Research Institute and PreCyte, Inc. If there are other authors, they declare that they have no known competing financial interests or personal relationships that could have appeared to influence the work reported in this paper.

## Data Availability

All data produced in the present work are either contained in the manuscript or supplementary tables and figures except for complete Raw and processed RNAseq data. The raw and processed RNAseq data discussed in this publication have been deposited in NCBI's Gene Expression Omnibus [1] (Edgar et al., 2002) and are accessible through GEO Series accession number GSE275699 (https://www.ncbi.nlm.nih.gov/geo/query/acc.cgi?acc=GSE275699). The R code used to train and evaluate the final classifiers, including feature selection and cross-validation procedures, is available at a public GitHub repository (https://github.com/jenjsmith/lung-cancer-iCAP-code.git).
